# ABHD6 and MAGL control 2-AG levels in the PAG and allodynia in a CSD-induced periorbital model of headache

**DOI:** 10.3389/fpain.2023.1171188

**Published:** 2023-05-23

**Authors:** Erika Liktor-Busa, Aidan A. Levine, Seph M. Palomino, Simar Singh, Jared Wahl, Todd W. Vanderah, Nephi Stella, Tally M. Largent-Milnes

**Affiliations:** ^1^ Department of Pharmacology, University of Arizona, Tucson, AZ, United States; ^2^ Department of Pharmacology, University of Washington, Seattle, WA, United States; ^3^ Department of Psychiatry and Behavioral Sciences, University of Washington, Seattle, WA, United States

**Keywords:** 2-AG, endocannabinoid, migraine, MAGL, ABHD6, PAG

## Abstract

**Introduction:**

The high prevalence and severe symptoms of migraines in humans emphasizes the need to identify underlying mechanisms that can be targeted for therapeutic benefit. Clinical Endocannabinoid Deficiency (CED) posits that reduced endocannabinoid tone may contribute to migraine development and other neuropathic pain conditions. While strategies that increase levels of the endocannabinoid n-arachidonoylethanolamide have been tested, few studies have investigated targeting the levels of the more abundant endocannabinoid, 2-arachidonoylgycerol, as an effective migraine intervention.

**Methods:**

Cortical spreading depression was induced in female Sprague Dawley rats via KCl (potassium chloride) administration, followed by measures of endocannabinoid levels, enzyme activity, and neuroinflammatory markers. Efficacy of inhibiting 2-arachidonoylglycerol hydrolysis to mitigate periorbital allodynia was then tested using reversal and prevention paradigms.

**Results:**

We discovered reduced 2-arachidonoylglycerol levels in the periaqueductal grey associated with increased hydrolysis following headache induction. Pharmacological inhibition of the 2-arachidonoylglycerol hydrolyzing enzymes, *α/β*-hydrolase domain-containing 6 and monoacylglycerol lipase reversed and prevented induced periorbital allodynia in a cannabinoid receptor-dependent manner.

**Discussion:**

Our study unravels a mechanistic link between 2-arachidonoylglycerol hydrolysis activity in the periaqueductal grey in a preclinical, rat model of migraine. Thus, 2-arachidonoylglycerol hydrolysis inhibitors represent a potential new therapeutic avenue for the treatment of headache.

## 1. Introduction

Migraine is a neurological pain disorder that affects more than 38 million Americans and is characterized by severe headache and sensory hypersensitivities (1). One third of patients with migraine experience aura, which is associated with the neurological event cortical spreading depression (CSD). CSD is a self-propagating wave of neuronal hyperexcitability followed by transient depression (2). In addition, CSD events induce neuroinflammation and have been shown to activate the periaqueductal gray (PAG) (3, 4). Despite severe symptoms, high prevalence, and pronounced neuroinflammation, current migraine interventions offer limited therapeutic benefit with low tolerability (5), highlighting the need to develop new treatment strategies.

The endocannabinoid (eCB) system plays a fundamental role in both central and peripheral pain processing (6). Two main eCB lipids, N-arachidonylethanolamine (AEA) and 2-arachidonoylglycerol (2-AG), are produced by N-acyl phosphatidylethanolamine phospholipase D (NAPE-PLD) and diacylglycerol lipases (DAGLs), respectively. While AEA is predominantly hydrolyzed by fatty acid amide hydrolase, 2-AG is hydrolyzed by *α*/*β*-hydrolase domain-containing 6 (ABHD6) and monoacylglycerol lipase (MAGL) (7). Both AEA and 2-AG activate the G protein-coupled cannabinoid receptors, CB_1_R and CB_2_R, which are implicated in synaptic relay and immune processes respectively (8).^,^

Mounting evidence supports the theoretical construct of Clinical Endocannabinoid Deficiency (CED) by which decreased AEA and 2-AG tone plays a role in functional pain disorders, including migraine (9, 10). These observations led to evaluation of the AEA role in migraine (9), yet no agents have been approved for clinical use. Our recent work showed that 2-AG levels are reduced in the periaqueductal grey (PAG) in a rat model of medication overuse headache. Furthermore, DAGL*α* inhibition induces periorbital allodynia at times when 2-AG levels in the PAG and other cortical regions associated with CSD were reduced (11). Based on this premise and work suggesting MAGL is implicated in a nitroglycerine model of migraine (12, 13), studies herein examined the influence of CSD induction on AEA and 2-AG levels in distinct brain areas, including V1M cortex, occipital visual motor cortex 1; PAG, periaqueductal gray; Vc, trigeminal nucleus caudalis; and TG, trigeminal ganglia. The occipital cortex (V1M) is the site of CSD induction which has inputs to PAG providing an anatomical link between CSD and the PAG. It is also known that PAG can both inhibit and facilitate nociception. The involvement of trigeminal system (Vc and TG) in regulation of migraine is widely described. Our work also investigated the role for DAGL*α*, MAGL, and ABHD6 to 2-AG regulation within the PAG, and the validity of increasing 2-AG signaling as a therapeutic approach for headache-like pain.

## 2. Materials and methods

### 2.1. Drugs and reagents

Ketamine/xylazine was purchased from Sigma-Aldrich (St. Louis, MO) and isoflurane from VetOne. (IL, USA). MJN110, KT-182, SR141716 (Rimonabant), and SR144528 were purchased from Cayman Chemicals (Ann Arbor, MI). All other chemicals, unless noted were purchased from Sigma-Aldrich (St. Louis, MO).

### 2.2. Animals

Intact, female Sprague Dawley rats (200–250 g, *n* = 345) were purchased from Envigo (Indianapolis, IN) and housed in a climate-controlled room on a 12/12 h light/dark cycle with lights on at 7:00 am with food and water available *ad libitum*. After arrival to the vivarium, rats were habituated for 1 week during which handling occurred daily to minimize stress. Animals were housed 3 per cage on arrival and individually after cannulation. All procedures were performed during the 12 h light cycle, according to the policies and recommendations of the International Association for the Study of Pain, NIH guidelines for laboratory animals, and with IACUC approval from the University of Arizona (# 17-223). Justification for animal numbers was consistent with NIH policy (NOT-OD-15-102), and experiments were randomized to blinded treatment groups to give 80% power to detect a treatment effect size of 20% compared to a baseline response of 5% at a significance level of 0.05 (14, 15). Female rats were used as headache disorders affect females to males at a nearly 3:1 ratio (16). The stage of estrous cycle was not determined in this study, since our former work indicated no changes in 2-AG level of selected brain areas during estrus cycle (17). The animal well-being and signs of distress were monitored daily after surgery for at least 5 days. A total of 29 animals were excluded based on predetermined criteria including identification as statistical outliers using GraphPad (*n* = 11), low post-surgical baseline allodynia values (*n* = 8), and temperature variation below IACUC standards mid-testing (*n* = 10).

### 2.3. Dural cannulation

Dural cannulation was performed as previously described (18). Briefly, anesthesia was induced with intraperitoneal 45:5:2 mg/kg cocktail of ketamine:xylazine:acepromazine. Rats were placed in a stereotactic frame (Stoelting Co.), and a 1.5- to 2-cm incision was made above the skull. A 0.66- to 1-mm hole (−6 mm A/P, −3 mm M/l from bregma) was made with a hand drill (DH-0 Pin Vise; Plastics One) to expose the dura. A guide cannula (0.5 mm from top of skull, 22 GA, #C313G; Plastics One) was inserted into the hole and sealed into place. Two 1 mm holes were made for stainless-steel screws (#MPX-080-3F-1M; Small Parts), and dental acrylic fixed the cannula to the screws and skull. A dummy cannula (#C313DC; Plastics One) was inserted to ensure patency of the guide cannula. Rats recovered over 6–8 d. Cannula placement and dural integrity at screw placement was confirmed postmortem.

### 2.4. Cortical injections

Cortical injections were performed using a Hamilton injector (30 GA, #80308 701 SN, Hamilton Company) customized to project 1.0 mm into the beyond the dura above the occipital cortex (V1M). At *t* = 0 min, filtered KCl (0.5 µl, 1 M) or artificial CSF [aCSF; 145 mM NaCl, 2.7 mM KCl, 1 mM MgCl_2_, 1.2 mM CaCl_2_, and 2 mM Na_2_HPO_4_ (pH 7.4)] was locally injected into V1M.

### 2.5. Pre- or post-cortical injection treatments

MJN110 (10 mg/kg, IP) was injected before (*t* = −30 min) or after (*t* =  +30 min) cortical injection of KCl. KT-182 (2 mg/kg, IP) was injected before (*t* = −3 h) or after (*t* =  +30 min) cortical injection of KCl according to their individual kinetics. SR141716 (1 mg/kg, IP) or SR144528 (1 mg/kg, IP) were administered 10 min before MJN110 or KT-182 in receptor dependency studies. MJN110, SR141716, and SR144528 were dissolved in DMSO: Tween80:saline (1:1:8, v/v/v). KT-182 was dissolved in ethanol-cremaphor-saline (1:1:18, v/v/v). The number of animals used in each experiment is indicated in Results section.

### 2.6. Periorbital mechanical allodynia

Periorbital allodynia was evaluated before and after cortical injection (*t* = 30, 60, 90, 120, 180, 360 min, and 24 h) by an observer blinded to drug condition. Rats were grouped based on their postsurgical baseline to ensure equivalent calculated pre-injection thresholds (6–8 g); rats exhibiting post-cannulation mechanical thresholds <6 g were removed from the study. Rats were acclimated to testing box then calibrated von Frey filaments (4.08–4.93 g) were applied perpendicularly to the midline of the forehead at the level of the eyes with enough force to cause the filament to slightly bend while held for 5s as described (19). Response was indicated by a sharp withdrawal of the head, vocalization, or severe batting at the filament with attempts to bite it. The withdrawal threshold was determined using a modified version of the Dixon up-down method.

### 2.7. Tissue harvest

Rats were anesthetized as above, then transcardially perfused with ice cold 0.1 M phosphate buffer at physiological flow rates (3.1 ml/min). After decapitation, tissue samples (V1M cortex, occipital cortex; PAG, periaqueductal gray; Vc, trigeminal nucleus caudalis; and TG, trigeminal ganglia) harvested, flash frozen in liquid nitrogen and stored at −80°C until further use.

### 2.8. Quantification of 2-Ag and AEA by LC-MS

Samples (*n* = 3–4/group) for LC-MS were purified by organic solvent extraction, as described by Wilkerson et al. (20) On the day of processing, tissues were weighed and Dounce homogenized in 1 ml of chloroform/methanol (2:1 v/v) with phenylmethylsulfonyl fluoride (PMSF, 1 mM) to inhibit degradation by endogenous enzymes. Homogenates were mixed with NaCl (0.3 ml, 0.7% w/v), vortexed, and centrifuged for (3,200 × g, 10 min, 4°C). Aqueous phase plus debris were collected and extracted twice more with 0.8 ml of chloroform. Organic phases were pooled, and internal standard was added to each sample. Mixed internal standards were prepared by serial dilution of d^4^-AEA and d^5^–2-AG in acetonitrile to calibrate concentration calculations and ensure run variability is accounted for according to best practices (21). The organic solvents were evaporated under nitrogen gas; glycerol in methanol (6 μl, 30%) was added before evaporation. Dried samples were reconstituted with chloroform (0.2 ml) and mixed with 1 ml ice-cold acetone. Mixtures were then centrifuged (1,800 × g, 5 min, 4°C). The organic layer of each sample was collected and evaporated under nitrogen.

Analysis of 2-AG and AEA was performed on an Ultivo triple quadrupole mass spectrometer combined with a 1,290 Infinity II UPLC system (Agilent, Palo Alto, CA). The instrument was operated in electrospray positive mode with a gas temperature of 150°C at a flow of 5l/min, nebulizer at 15 psi, capillary voltage of 4,500 V, sheath gas at 400°C with a flow of 12l/min and nozzle voltage of 300 V. Transitions monitored were 348.3 → 287.3 and 62, 352.3 → 287.4 and 65.9, 379.3 → 287.2 and 269.2, and 384.3 → 287.2 and 296.1 for AEA, 2-AG, d^4^-AEA and d^5^-2-AG. The first fragment was used for quantification and the second fragment was used for confirmation. The first 3 min of analysis was diverted to waste. Chromatographic separation was achieved using an isocratic system of 21% 1 mM ammonium fluoride and 79% methanol on an Acquity UPLC BEH C-18 1.7u 2.1 × 100 mm column (Waters, Milford, MA) at 60°C. After each injection the column was washed with 90% methanol for one minute then re-equilibrated for 5 min prior to the next injection. Samples were maintained at 4°C. Mixed calibration solutions were prepared by serial dilution of AEA and 2-AG stock solutions in 80% C₂H₃N. Calibration curves were prepared for each analysis by adding 10 µl internal standard solution to 20 µl standard solution. 200 µl of 80:2° C₂H₃N:H_2_O was added to dried samples which were then vortexed and sonicated. Samples were centrifuged at (15,800 × g, 5 min, 4°C), supernatant transferred to autosampler vials and 5 µl was injected for analysis.

### 2.9. Membrane preparation

Flash-frozen tissue was thawed on ice, Dounce homogenized in ice cold lysis buffer (20 mM HEPES, 1 mM MgCl_2_, 2 mM DTT, 10 units/ml benzonase) and centrifuged at 100,000 × g for 45 min (Beckman Coulter rotor Ti55). The supernatant was discarded, and the pellet resuspended in buffer HEPES (20 mM) supplemented with DTT (2 mM). Protein concentration of samples was determined (DC protein assay, Bio-Rad) before aliquoting and flash freezing samples in liquid nitrogen. Samples were stored at −80 until further use.

### 2.10. Western- immunoblotting

Samples (*n* = 3–4/group) were thawed on ice, 25 µg of protein was loaded on a 10% pre-cast polyacrylamide gel (Mini-PROTEAN TGX™, Biorad) and then transferred to a PVDF membrane (Immobilon-PSQ, Sigma Aldrich). Membranes were blocked with 5% milk in tris-buffered saline with 0.05% TWEEN-20 (TBST) for 1 h at RT. Following blocking, membranes were incubated overnight at 4°C in primary antibodies (ABHD6: characterized by Deng, et al. (22); MAGL: ab77398, Abcam; Iba1: PA5-27436, Invitrogen; GFAP: ab68428, Abcam; *α*-tubulin: 3873S, Cell Signaling) in blocking buffer. The membranes were washed three times with TBST, incubated with secondary antibodies [goat-anti-mouse IgG-HRP and donkey anti-goat secondary IgG-HRP (Santa Cruz)] for 1 h at RT, then washed with TBST thrice. Membranes were incubated in enhanced chemiluminescent substrate (SuperSignal West Femto Maximum Sensitivity Substrate, ThermoFisher Scientific) for 60 s at RT and detected using Chemidoc MP (Bio-Rad). Membranes were stained with Ponceau S (G Biosciences) to obtain total protein. Analysis performed with ImageJ.

### 2.11. DAG and PGE_2_ ELISA

DAG ELISA kit (Aviva System Biology, OKEH02607) was used according to manufacturer's instruction. DAG: Tissue samples (*n* = 3–4/group) were weighed, homogenized in PBS buffer and stored overnight at ≤−20°C. Two freeze-thaw cycles were conducted to break the cell membranes. Homogenates were centrifuged (5,000 g, 10 min, 4°C). 5 µl of the supernatant was applied in the immunoassay. PGE_2_ ELISA kit (Abcam, ab133021) was used according to manufacturer`s guidance. Tissue samples (*n* = 3–4/group) were weighed and homogenized in assay buffer supplemented with indomethacin (10 µM) to block endogenous prostaglandin synthetase. The homogenized samples were centrifuged (8,000 × g, 10 min, 4°C) and 100 µl of supernatant was used.

### 2.12. Statistical analysis

GraphPad Prism 7.0 and 8.3.1 (GraphPad Software) were used for statistical analysis. Numbers required to achieve statistical power (80% power to detect 20% difference) were determined by G.Power3.1 *a priori* with historical data in naïve male and female samples and confirmed *post hoc* with actual data collected. Unless otherwise stated, the data were expressed as mean ± S.E.M. Periorbital allodynia measurements were assessed using a repeated measure two-way ANOVA to analyze differences between treatment groups over time with a Bonferroni test applied *post hoc*. Molecular studies were compared by unpaired t-test or one-way ANOVA, as indicated. Differences were considered significant if *p* ≤ 0.05.

## 3. Results

### 3.1. Induction of CSD reduces 2-Ag levels and transiently upregulates ABHD6/MAGL expression in the PAG

**Figure 1:**
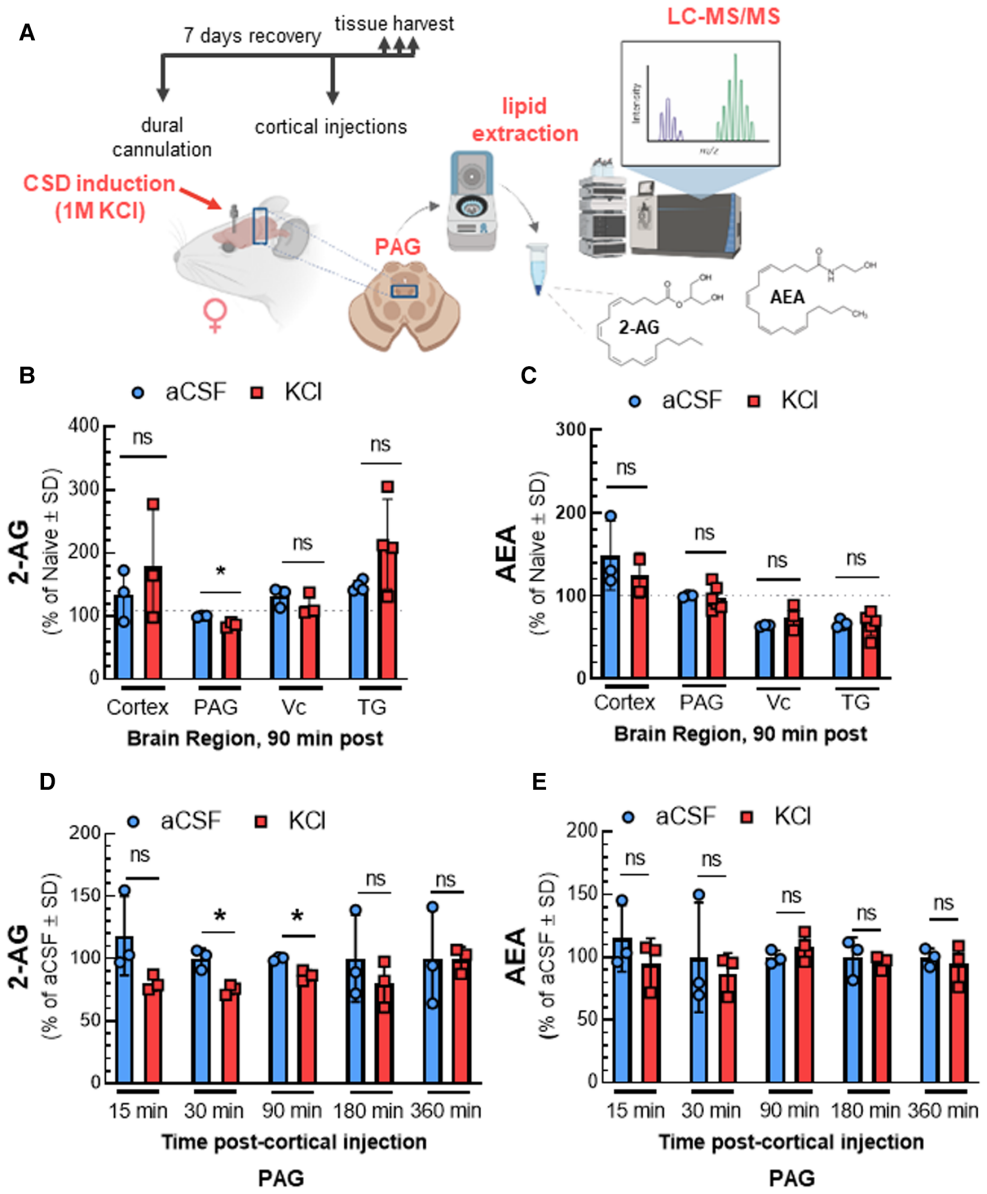
Time-dependent reduction of 2-AG levels in PAG after CSD in female rats. The surgery of dural cannulation was performed on female Sprague Dawley rats. After 7 days recovery period, a focal injection of 0.5 µl of 1 M KCl or artificial CSF (aCSF) were injected through the guide cannula into the cerebral cortex. Tissue samples (cortex, PAG, Vc, and TG) were collected at 90 min after cortical injections. In a separate set of experiment, PAG samples were harvested at different time-points (15, 30, 90, 180, and 360 min) after aCSF or KCl injection. The samples were subjected to LC-MS to quantify endocannabinoid levels. (**A**) Schematic outlining protocol to measure the changes in endocannabinoid levels of selected brain regions after CSD induction. (**B**) Reduced 2-AG level was observed only in PAG samples at 90 min time-point after CSD induction. There were no significant differences at 2-AG levels in cortex, Vc, and TG samples at 90 min after KCl injection. (2-AG: aCSF vs. KCl—cortex: *p* = 0.47, PAG: *p* = 0.023, Vc: *p* = 0.36, TG: *p* = 0.11, as assessed by multiple t-test). Values are expressed as % of naive ± SD (*n* = 3-6/condition). The dotted line represents the 2-AG levels in naïve samples. (**C**) Induction of CSD by cortical injection of KCl did not cause significant differences in AEA levels 90 min post-injection in cortex, PAG, Vc, and TG samples. (AEA: aCSF vs. KCl—cortex: *p* = 0.54, PAG: *p* = 0.79, Vc: *p* = 0.31, TG: *p* = 0.97, as assessed by multiple t-test). Values are expressed as % of naive ± SD (*n* = 3-6/condition). The dotted line represents the AEA levels in naïve samples. (**D**) Decreased levels of 2-AG were detected in PAG at 30 and 90 min after KCl injection as compared to aCSF (2-AG: aCSF vs. KCl—15 min: *p* = 0.17, 30 min: 0.019, 90 min: *p* = 0.020, 180 min: *p* = 0.44, 360 min: *p* = 0.98 as assessed by two-way ANOVA with Bonferroni post-test, Time × Injection: F(4,16) = 0.7187). AEA levels were not significantly different between aCSF and KCl groups at any time point analyzed (**E**). (AEA: aCSF vs. KCl—15 min: *p* = 0.36, 30 min: *p* = 0.33, 90 min: *p* = 0.33, 180 min: *p* = 0.70, 360 min: *p* = 0.65 as assessed by two-way ANOVA with Bonferroni post-test, Time × injection: F(1.648,7.827) = 2.096). Values are expressed as % of aCSF ± SD (*n* = 3-6/condition). ns = non-significant. *p < 0.05 aCSF vs. KCl.

We previously showed that acute DAGL*α* inhibition reduces 2-AG levels in the PAG at times of peak periorbital allodynia, suggesting a role for 2-AG in the induction of headache like pain (11). Here we investigated whether CSD induction by cortical injection of KCl similarly reduced 2-AG as compared to control aCSF injection (Figure 1A) by measuring 2-AG and AEA in the V1M cortex, PAG, Vc, and TG via LC-MS from tissue harvested 90 min after cortical injection. Figure 1B shows that CSD induction decreased 2-AG levels at 90 min within the PAG but not in cortex, Vc, and TG (2-AG: aCSF vs. KCl—cortex: *t*(4) = 0.8003, *p* = 0.47, PAG: *t*(4) = 5.3366, *p* = 0.023, Vc: *t*(4) = 1.033, *p* = 0.36, TG: *t*(6) = 1.849, *p* = 0.11, Student's t-test). AEA levels were unchanged by CSD induction in the selected brain regions (Figure 1C; AEA: aCSF vs. KCl—cortex: *t*(3) = 0.6971, *p* = 0.536, PAG: *t*(7) = 0.2765, *p* = 0.790, Vc: *t*(4) = 1.170, *p* = 0.307, TG: *t*(7) = 0.04016, *p* = 0.969, Student's t-test). Since we observed significant changes in 2-AG levels only in PAG, subsequent experiments focused on that region.

PAG samples were analyzed after CSD induction to determine the time course of 2-AG decreases. Reduced 2-AG levels were observed 30 min after cortical KCl injection and remained significantly decreased relative to aCSF until 180 min post injection [Figure 1D; 2-AG: two-way ANOVA, F(4,16) = 0.7187; aCSF vs. KCl—15 min: *p* = 0.17, 30 min: 0.019, 90 min: *p* = 0.020, 180 min: *p* = 0.44, 360 min: *p* = 0.98, Bonferroni post-test]. No significant changes in AEA level were detected in any time-point (Figure 1E). These data show significant region and time selective reductions in 2-AG tone in the PAG after CSD induction.

**Figure 2:**
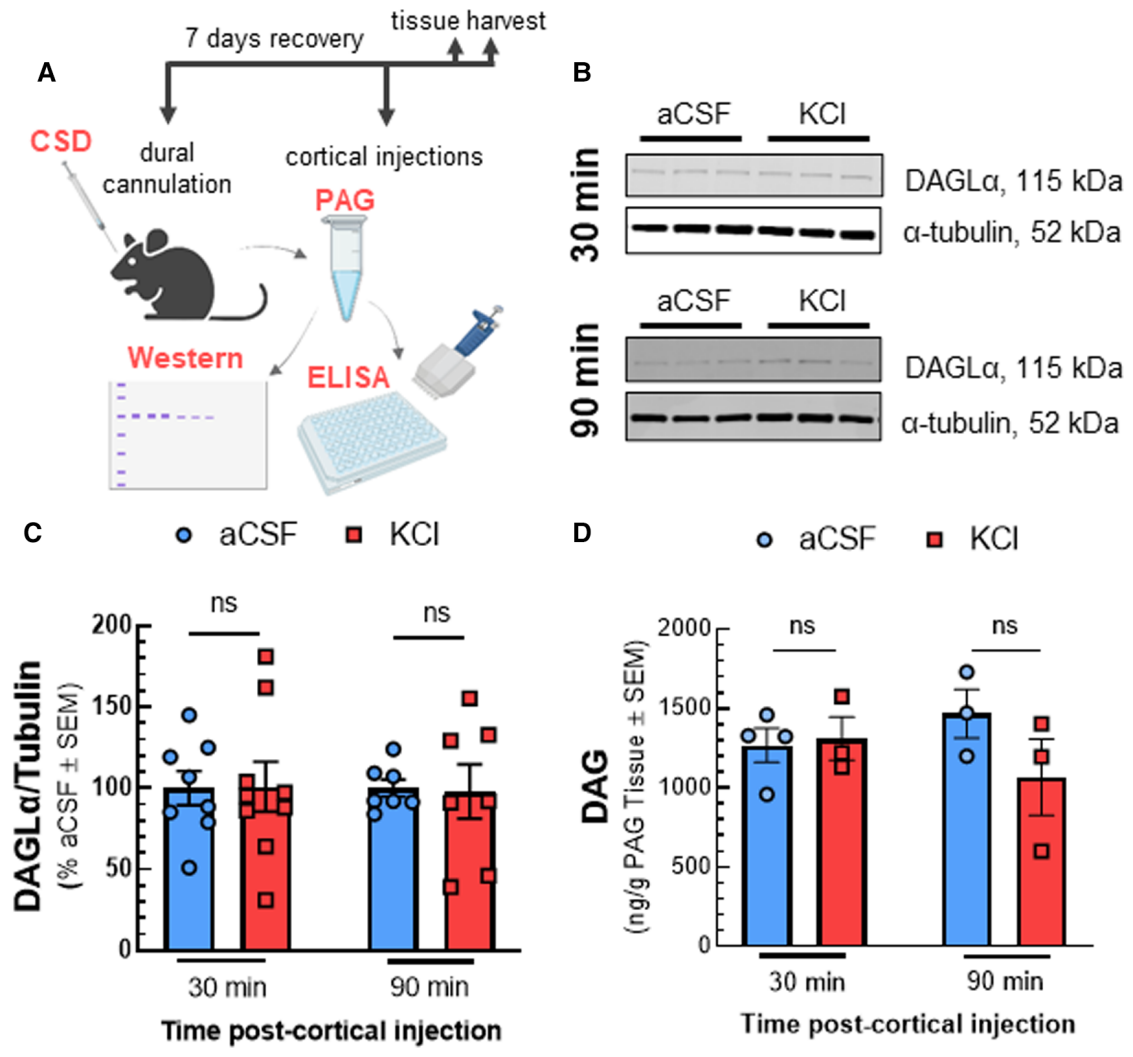
Reduced 2-AG in the PAG is not linked to decreased enzymatic synthesis by DAGL. Dural cannula was surgically implanted to female Sprague Dawley rats. After recovery period, PAG samples were harvested at different time-points (30 and 90 min) after cortical injection of KCl (0.5 µl, 1M) or aCSF (0.5 µl), then subjected to Western-immunoblotting and DAG ELISA. (**A**) Schematic of measuring the expression of DAGL and DAG level in PAG samples after CSD induction. (**B**) Representative immunoblots indicating DAGL*α* and *α*-tubulin as a loading control in PAG samples harvested at 30 and 90 min after CSD induction or aCSF injection. (**C**) Cortical KCl did not significantly change DAGL*α* expression as compared to aCSF control at times when 2-AG are reduced. All data represent the % of aCSF relative expression of DAGL*α* ± SD (*n* = 3-4). KCl vs. aCSF ns = non-significant, F time × treatment (3,28) = 2.169, *p* = 0.11 as assessed by two-way ANOVA with Bonferroni post-test. (**D**) There was no significant difference in the level of DAG, the precursor of 2-AG in PAG samples harvested at 30 and 90 min after cortical injection of KCl as compared to aCSF controls. Data show the average amount of DAG precursor (pg/g) in PAG samples ± SD (*n* = 3–4). KCl vs. aCSF ns = non-significant, F time × treatment(1,9) = 1.914, *p* = 0.20 as assessed by two-way ANOVA with Bonferroni post-test.

**Figure 3:**
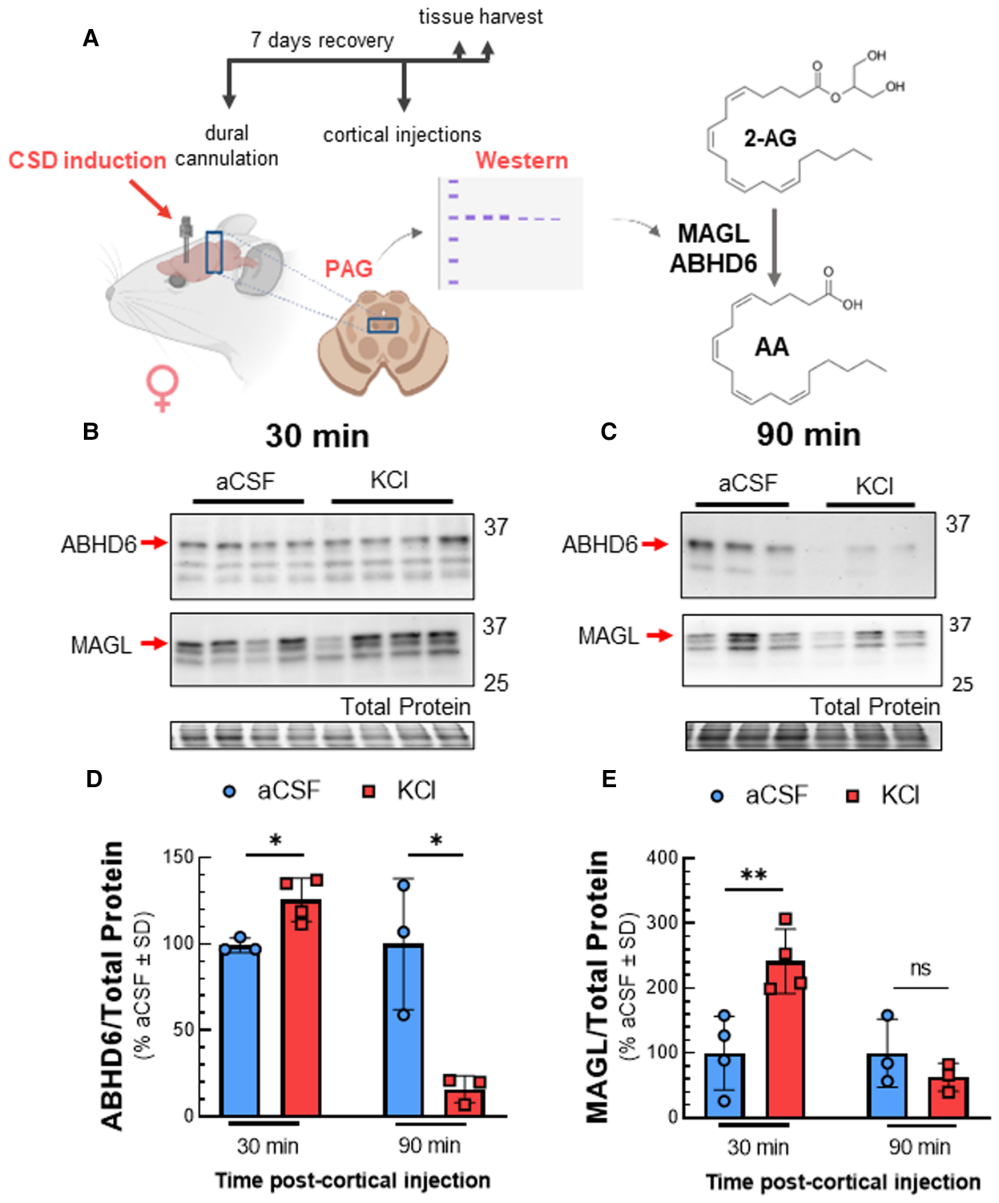
Total expression of ABHD6 and MAGL in PAG after cortical KCl injection. Female Sprague Dawley rats were injected with KCl (0.5 µl, 1M) or aCSF (0.5 µl) through the guide cannula one week after dural cannulation surgery. PAG samples obtained 30 and 90 min after cortical injections were subjected to Western immunoblotting to determine expression of ABHD6 and MAGL. (**A**) Schematic of experimental setting to detect changes in the expression of MAGL and ABHD6 in CSD induced model. (**B**) Representative immunoblots showing the detection of MAGL and ABHD6 along with total protein in PAG samples 30 min after cortical injection of KCl or aCSF. (**C**) Representative immunoblots of PAG samples showing the detection of MAGL and ABHD6 with total protein 90 min after CSD induction. (**D**) Cortical KCl significantly increased ABHD6 detection 30 min after injection however the detection of ABHD6 was significantly reduced at the 90 min time-point (unpaired *t*-test, 30min: *t*(5) = 3.44, *p* = 0.018; 90 min: *t*(4) = 3.752, *p* = 0.020) (**E**) MAGL detection was increased 30 min post-injection as compared to cortical injection of aCSF at times when 2-AG are reduced, but no significant difference was observed at 90 min in PAG samples (unpaired *t*-test, 30 min: *t*(6) = 3.762, *p* = 0.0094; 90 min: *t*(4) = 1.133, *p* = 0.32). Values are % of aCSF control ± SD (*n* = 3/condition) ns = non-significant (*p* > 0.05), *p < 0.05, **p < 0.01.

Reductions in 2-AG tone may result from decreased synthesis by DAGL*α* or increased degradation by MAGL and/or ABHD6 (8). Western blot analysis of PAG tissue after KCl injection showed that DAGL detection in PAG tissue was not affected by CSD [Figures 2B,C; two-way ANOVA, F(3,28) = 2.169, *p* = 0.11]. Likewise, levels of DAG, the substrate for DAGL, were unaffected by cortical KCl injection [Figure 2D; two-way ANOVA, F(1,9) = 1.914, *p* = 0.20]. In contrast, ABHD6 and MAGL detection increased 30 min after cortical injection of KCl as compared to aCSF control and were both reduced below basal levels at 90 min (Figures 3B–E; MAGL: 30 min: *t*(6) = 3.762, *p* = 0.0094; 90 min: *t*(4) = 1.133, *p* = 0.32; ABHD6: 30min: *t*(5) = 3.44, *p* = 0.018; 90 min: *t*(4) = 3.752, *p* = 0.020; unpaired Student t-test). Thus, decreases in PAG 2-AG levels triggered by CSD do not reflect decreased DAGL*α* function but suggest a transient increase in total expression of MAGL/ABHD6.

### 3.2. CSD triggered changes in PAG neuroinflammatory response

**Figure 4:**
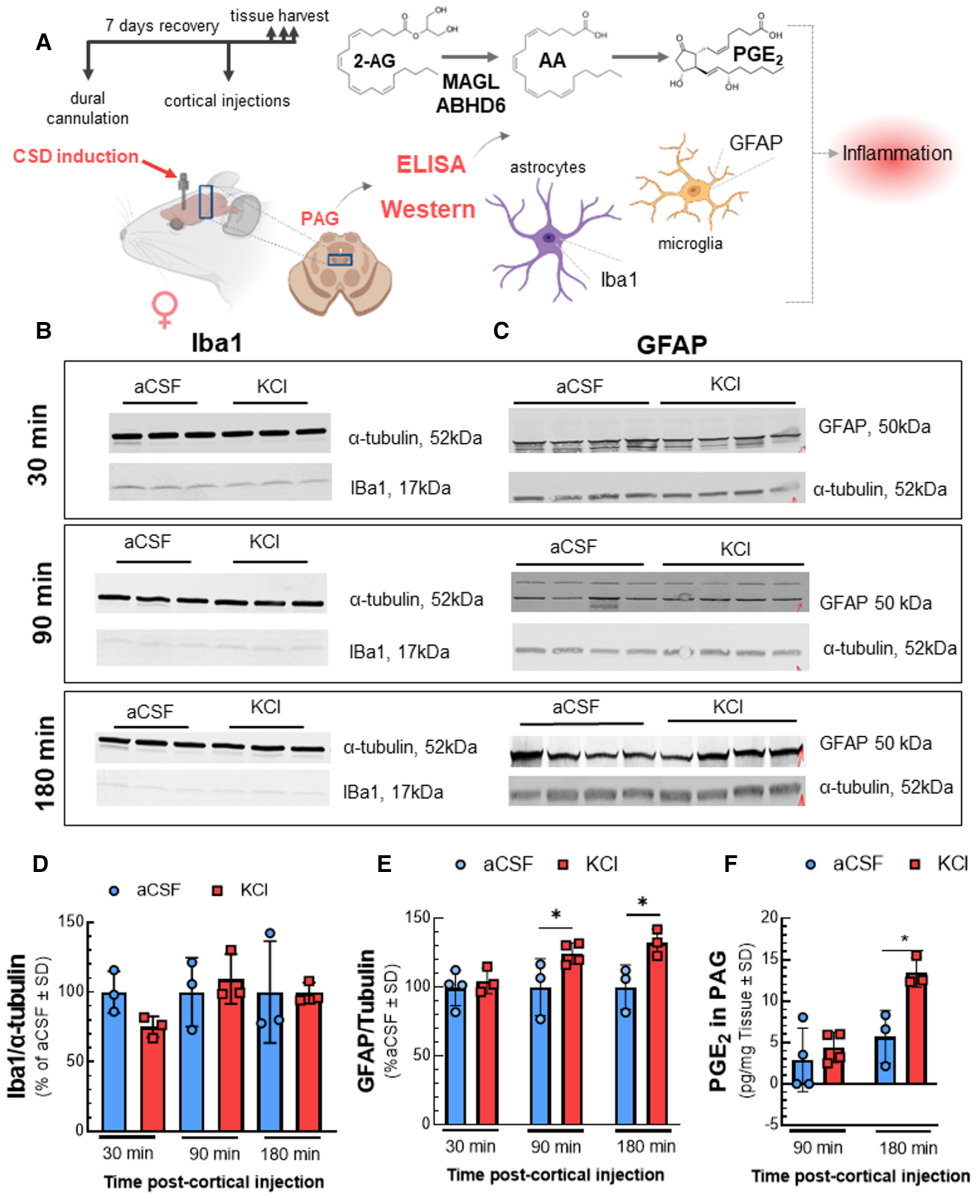
Elevated neuroinflammatory biomarkers in PAG after cortical KCl injection. Dural cannulation was performed on female Sprague Dawley rats. After 7-days recovery, focal injection of 0.5 µl of 1 M KCl or artificial CSF (aCSF) were delivered through the dural cannula into the cerebral cortex. PAG samples were harvested at three different timepoints (30, 90, and 180 min) post aCSF or KCl injections and subjected to Western immunoblotting or ELISA. Western immunoblotting was performed to detect the expression of IBa1 and GFAP. The level of PGE2 was measured by ELISA assay. (**A**) Schema of measuring the changes of inflammatory factors in PAG samples after CSD induction. (**B**) Representative images of PAG samples showing the detection of Iba1 along with *α*-tubulin, as loading control at 30, 90, and 180 min time-points after cortical injections. (**C**) Representative images showing the detection of GFAP with *α*-tubulin, as loading control in PAG samples harvested at 30-, 90-, and 180-min time-points after CSD induction. (**D**) Relative detection of Iba1 in PAG was not significantly different at 30, 90, and 180 min after injection of KCl compared to aCSF control. Data represent % of aCSF control ± SD (*n* = 3-4/condition; two-way RM ANOVA time × cortical injection F(2,8) = 3.369, *p* = 0.087). (**E**) The detection of GFAP was elevated at 90 and 180 min post-KCl compared to aCSF control [two-way RM ANOVA F(1,6) = 8.773, Bonferroni post-test: 90 min *p* = 0.025; 180 min *p* = 0.044]. Data represent % of aCSF control ± SD (*n* = 3–4/condition). (**F**) Increased level of PGE2 was observed in PAG 180 min after cortical KCl injection (two-way RM ANOVA time × cortical injection F(1,8) = 10.08, Bonferroni post-test *p* = 0.015). Values are mean ± SD (*n* = 3–4/condition). *p < 0.05.

As cortical neuroinflammation is reported during CSD (23), the endocannabinoid system plays a role in mitigating neuroinflammation (8), and degradation of 2-AG and AEA generate proinflammatory mediators (24), we used WB to detect activation of astrocytes and microglia using GFAP and Iba1, respectively, and measured PGE_2_ by ELISA in the PAG (Figure 4). While Iba1 detection in the PAG was not affected by cortical KCl injection [Figures 4B,D; two-way ANOVA F(5,12) = 0.9212; 30 min: *p* = 0.6914, 90 min: *p* = 0.9922, 180 min: *p* > 0.9999, Bonferroni], GFAP detection increased after 90 min and remained elevated at 180 min [Figures 4C,E; two-way ANOVA F(1,6) = 8.773; 30 min: *p* > 0.999, 90 min: *p* = 0.025, 180 min: *p* = 0.044, Bonferroni]. PGE_2_ levels in the PAG were increased 2.4-fold over aCSF by 180 min [Figure 4F; two-way ANOVA, F(1,8) = 10.08; 180 min: *p* = 0.015, Bonferroni]. Thus, CSD induction is associated with a time-dependent neuroinflammatory response within the PAG characterized by reactive gliosis and production of PGE_2_ at times after 2-AG is decreased. An increase in PGE_2_ levels is consistent with 2-AG hydrolysis, therefore subsequent experiments evaluated the role of ABHD6 and MAGL in CSD associated headache-like pain.

### 3.3. ABHD6 inhibition prevents and reverses CSD induced periorbital allodynia in cannabinoid receptor-independent and -dependent manners, respectively

**Figure 5:**
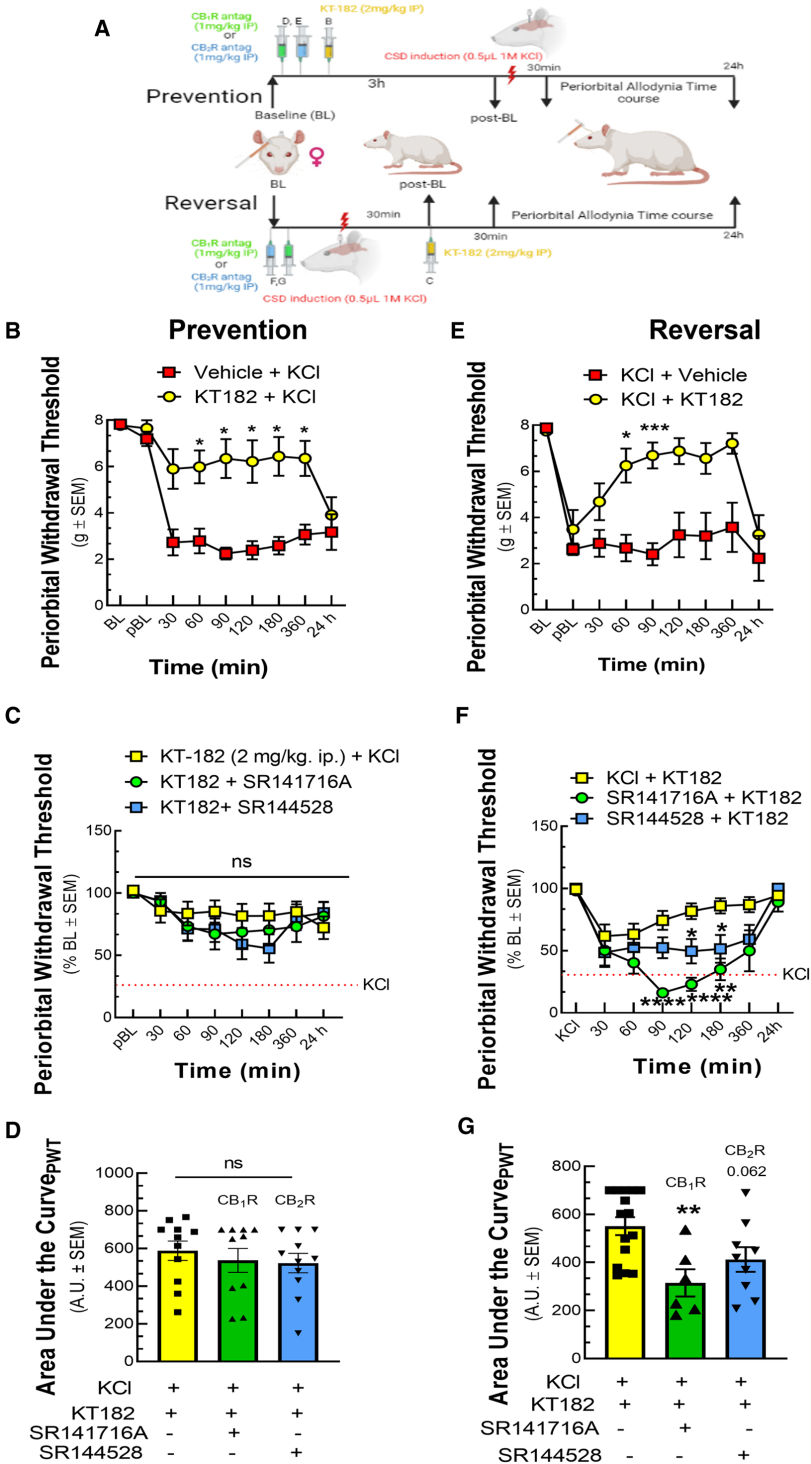
ABHD6 inhibition prevented and reversed periorbital allodynia in a variable CBR dependent manner. Female Sprague Dawley rats were utilized for behavior assays 7 days after implantation of dural canula. The animals were injected with cortical KCl (0.5 µl, 1M) in combination with KT182 (2 mg/kg, ip), an ABHD6 inhibitor or vehicle in prevention and reversal treatment paradigms. In the prevention paradigm, KT-182 was injected 3 h before the application of KCl. In the reversal paradigm, KT-182 was applied 30 min after cortical injection of KCl. In a separate set of experiment, CB_1_R and CB_2_R antagonist SR141716 (1 mg/kg, IP) or SR144528 (1 mg/kg, IP), respectively were administered 10 min before the injection of KT-182. Facial sensitivity was measured at baseline (BL), post-baseline (pBL), 30, 60, 90, 120, 180, 360 min, and 24 h after CSD induction by calibrated von Frey filaments. (**A**) Schema of the experimental timeline. (**B**) Injection of KT-182 significantly prevented CSD-induced periorbital allodynia at different time-points as compared to vehicle controls **(**two-way ANOVA (time × treatment) F(8,135) = 5.838, Bonferroni post-test: 30 min: *p* = 0.081; 60 min: *p* = 0.026, 90 min: *p* = 0.013; 120 min: *p* = 0.033, 180 min: *p* = 0.018, 360 min: *p* = 0.027) (**C**) Neither SR144528 nor SR141716 significantly blocked this effect. (**D**) AUC analysis of treatment groups confirmed no loss of effect with SR141716 or SR144528. (**E**) Injection of KT182 in the reversal treatment paradigm alleviated periorbital allodynia caused by cortical KCl at 60 and 90 min (two-way ANOVA (time × treatment) F(8,120) = 4.032, Bonferroni post-test: 60 min: *p* = 0.014, 90 min: *p* = 0.0003, 120 min: *p* = 0.079, 180 min: *p* = 0.16, 360 min: *p* = 0.12) (**F**) ABHD6 reversal of CSD periorbital allodynia was blocked by SR144528 and SR141716 within the 90-180 min (two-way ANOVA (time × treatment) F(14,186) = 4.083, Bonferroni post-test: KCl + KT-182 vs. KCl + KT-182+ SR141716: 90 min: *p* < 0.0001, 120 min: *p* < 0.0001, 180 min *p* = 0.0018; KCl + KT-182 vs. KCl + KT-182 + SR144528: 90 min: *p* = 0.1017, 120 min: *p* = 0.0359, 180 min: *p* = 0.044. (**G**) AUC analysis of treatment groups was significantly decreased for the SR141716 group, indicating CB_1_R dependence [KCl + KT-182 vs. KCl + KT-182+ SR141716: *p* = 0.0048, one-way ANOVA, F(2,27) = 6.328, Dunnett]. Values are the mean ± SEM (*n* = 9/condition). ns = non-significant, *p < 0.05, **p < 0.01.

Using the brain-penetrant ABHD6 inhibitor, KT-182, the next study examined the role of ABHD6 in induction and maintenance of CSD-associated periorbital allodynia. Pretreatment with KT-182 (2 mg/kg, 3 h) prevented the development of periorbital allodynia starting 60 min after cortical KCl injection and lasting 360 min [Figure 5B; two-way ANOVA F(8,135) = 5.839; 60–360 min: *p* < 0.05, Bonferroni, *n* = 8–11/group]. Neither the CB_1_R (SR141716, 1 mg/kg; Figure 5C, *n* = 10–11/group) nor the CB_2_R antagonist (SR144528, 1 mg/kg; Figure 5C) blocked the preventative effect of KT-182 [AUC analysis: KCl + KT-182 vs. KCl + KT-182 + SR141716: *p* = 0.7426; KCl + KT-182 vs. KCl + KT-182 + SR144528: *p* = 0.6112, one-way ANOVA, F(2,29) = 0.3970, Dunnett, Figure 5D]. In the reversal paradigm, KT182 (2 mg/kg, *t *= +30 min) reversed the established periorbital allodynia 60 min post cortical KCl injection [Figure 5E; two-way ANOVA, F(8,120) = 4.032; 60 min: *p* = 0.014, 90 min: *p* = 0.0003, Bonferroni, *n* = 7–10/group]. KT-182 reversal of periorbital allodynia was attenuated by both CB_1_R and CB_2_R antagonism between 90 and 180 min [Figure 5F; two-way ANOVA, F(14,186) = 4.083; KCl + KT-182 vs. KCl + KT-182 + SR141716, 90–180 min: *p* < 0.01, Bonferroni; KCl + KT-182 vs. KCl + KT-182 + SR144528, 120–180 min: *p* < 0.05, Bonferroni, *n* = 6–12/group]. AUC analysis revealed the dominating effect was by CB_1_R [Figure 5G; KCl + KT-182 vs. KCl + KT-182+ SR141716: *p* = 0.0048; KCl + KT-182 vs. KCl + KT-182 + SR144528: *p* = 0.0623, one-way ANOVA, F(2,27) = 6.328, Dunnett]. These data show that ABHD6 plays a time-dependent role in inducing CSD-associated periorbital allodynia independent of CB_1_R/CB_2_R whereas maintenance of headache-like responses occur via a CB_1_R > CB_2_R-dependent mechanism.

### 3.4. MAGL inhibition prevents and reverses CSD induced periorbital allodynia in a CB_2_R dependent manner

**Figure 6:**
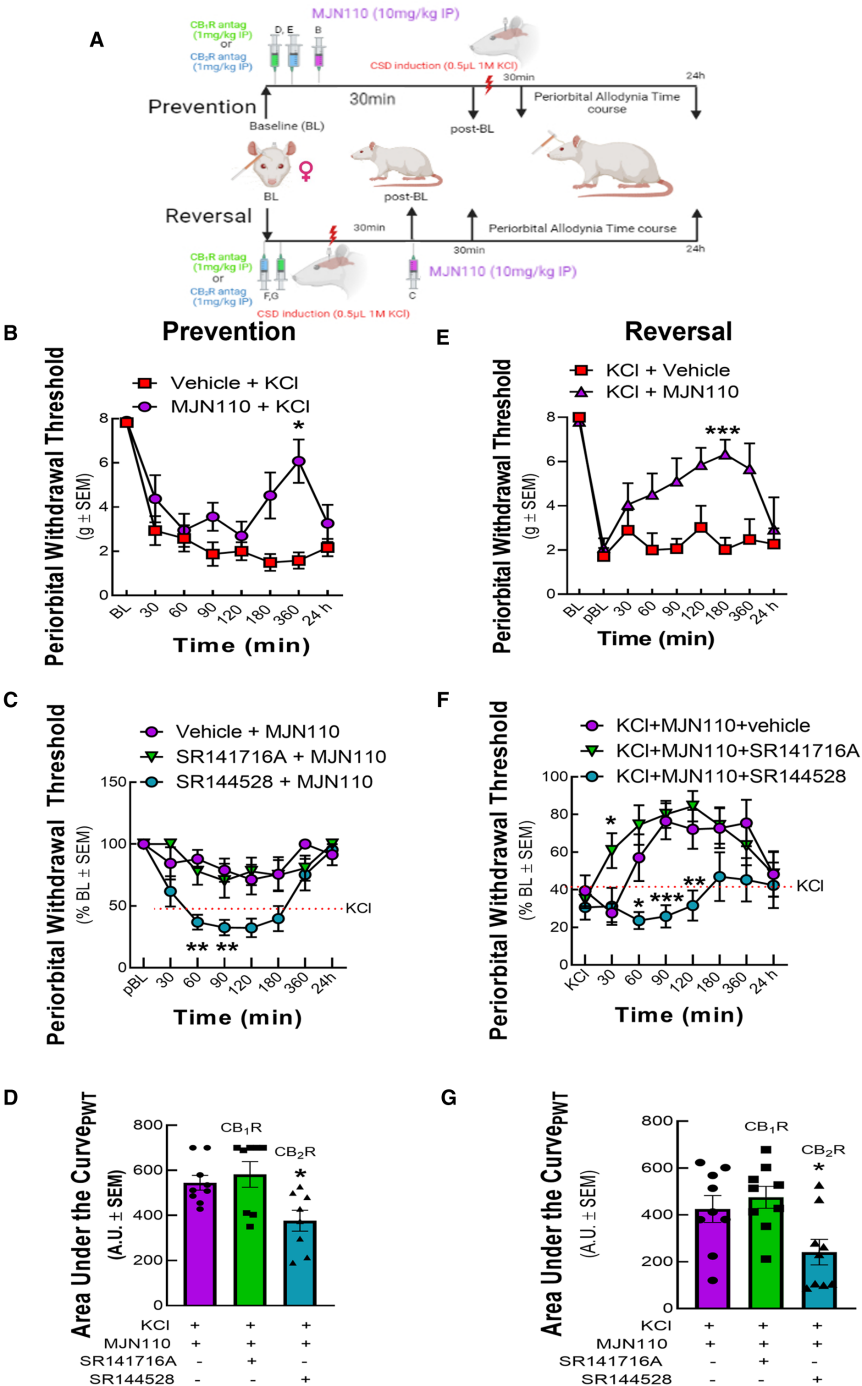
MAGL inhibition prevented and reversed periorbital allodynia induced by cortical KCl injection in a CB_2_R dependent manner. Dural cannulation was performed on female Sprague Dawley rats. After the recovery period, MJN110 (10 mg/kg) was injected intraperitoneally (IP) 30 min before (*t* = −30 min) for the prevention paradigm or 30 min after (*t *= +30 min) cortical injection of KCl for the reversal paradigm. In a separate set of experiment, CB_1_R and CB_2_R antagonist SR141716 (1 mg/kg, IP) or SR144528 (1 mg/kg, IP), respectively were administered 10 min before the injection of MJN110. Facial sensitivity was measured at baseline (BL), post-baseline (pBL), 30, 60, 90, 120, 180, 360 min, and 24 h after CSD induction by calibrated von Frey filaments. (**A**) Schema of the experimental timeline. (**B**) Administration of MAGL enzyme inhibitor, MJN110 before CSD induction significantly increased periorbital withdrawal threshold at 360 min time-point [two-way ANOVA F(7,77) = 3.604, Bonferroni post-test: 360 min: *p* = 0.017]. (**C**) The CB_2_R inverse agonist, SR144528 significantly blocked the preventative effects of MJN110 at the 60 and 90 min timepoints [two-way ANOVA F(14,126) = 3.345, Bonferroni post-test: 60 min Veh vs. SR144528, *p* = 0.0014; 90 min Veh vs. SR144528, *p* = 0.01]. (**D**) AUC analysis of treatment groups confirmed loss of effect for the SR144528 group, indicating CB_2_R dependence [Prevention AUC: KCl + MJN110 vs. KCl + MJN110+ SR141716: *p* = 0.7927; KCl + MJN110 vs. KCl + MJN110 + SR144528: *p* = 0.0278; one-way ANOVA F(2,22) = 5.620, Dunnett, *n* = 8/group]. (**E**) MAGL inhibition by MJN110 also mitigated headache-like pain after CSD induction compared to Veh controls [two-way ANOVA, F(8,92) = 2.911 *p* = 0.006, Bonferroni post-test: 60 min: *p* = 0.50; 90 min: *p* = 0.19, 120 min: *p* = 0.37, 180 min: *p* < 0.002; 360: *p* = 0.932]. (**F**) Dosing with the CB_2_R inverse agonist, SR144528, but not SR141716A blocked this effect at the 60, 90, and 120 min timepoints [two-way ANOVA, F(14,192) = 1.722, *p* = 0.0539, Bonferroni post-test: 60 min Veh vs. SR144528, *p* = 0.040; 90 min Veh vs. SR144528, *p* = 0.0008; 120 min Veh vs. SR144528, *p* = 0.0094]. (**G**) AUC analysis of treatment groups confirmed loss of effect for the SR144528 group, indicating CB_2_R dependence [Reversal AUC: KCl + MJN110 vs. KCl + MJN110+ SR141716: *p* = 0.7303; KCl + MJN110 vs. KCl + MJN110 + SR144528: *p* = 0.0405; one-way ANOVA F(2,24) = 5.388; Dunnett, *n* = 9/group]. Values are the mean ± SEM (*n* = 9/condition). ns = non-significant, *p < 0.05, **p < 0.01.

To determine the role of MAGL in CSD-associated periorbital allodynia, we used the brain penetrant MAGL inhibitor, MJN110. Treatment of rats with MJN110 (10 mg/kg) either 30 min before or 30 min after KCl injection prevented and reversed headache-like pain (Figures 6B,E). In the prevention paradigm, MJN110 induced in a shorter response that peaked at 360 min, whereas a longer response that peaked at 180 min was observed in the reversal paradigm (Figures 6B,E; Prevention, two-way ANOVA F(7,77) = 3.604; 360 min: *p* = 0.017, Bonferroni, *n* = 7–8/group; Reversal, two-way ANOVA, F(8,92) = 2.911; 180 min: *p* < 0.002, Bonferroni, *n* = 7–9/group). Pretreatment with SR144528 (1 mg/kg), but not with SR141716 (1 mg/kg) blocked both the prevention and reversal of periorbital allodynia mediated by MJN110 (Figures 6C,D,F,G; Prevention AUC: KCl + MJN110 vs. KCl + MJN110+ SR141716: *p* = 0.7927; KCl + MJN110 vs. KCl + MJN110 + SR144528: *p* = 0.0278; one-way ANOVA F(2,22) = 5.620, Dunnett, *n* = 8/group; Reversal AUC: KCl + MJN110 vs. KCl + MJN110+ SR141716: *p* = 0.7303; KCl + MJN110 vs. KCl + MJN110 + SR144528: *p* = 0.0405; one-way ANOVA F(2,24) = 5.388; Dunnett, *n* = 9/group). Thus, MAGL plays a role in CSD periorbital allodynia induction and maintenance. Blockade of MAGL both prevents and reverses CSD-associated allodynia in a CB_2_R-dependent manner.

## 4. Discussion

**Figure 7:**
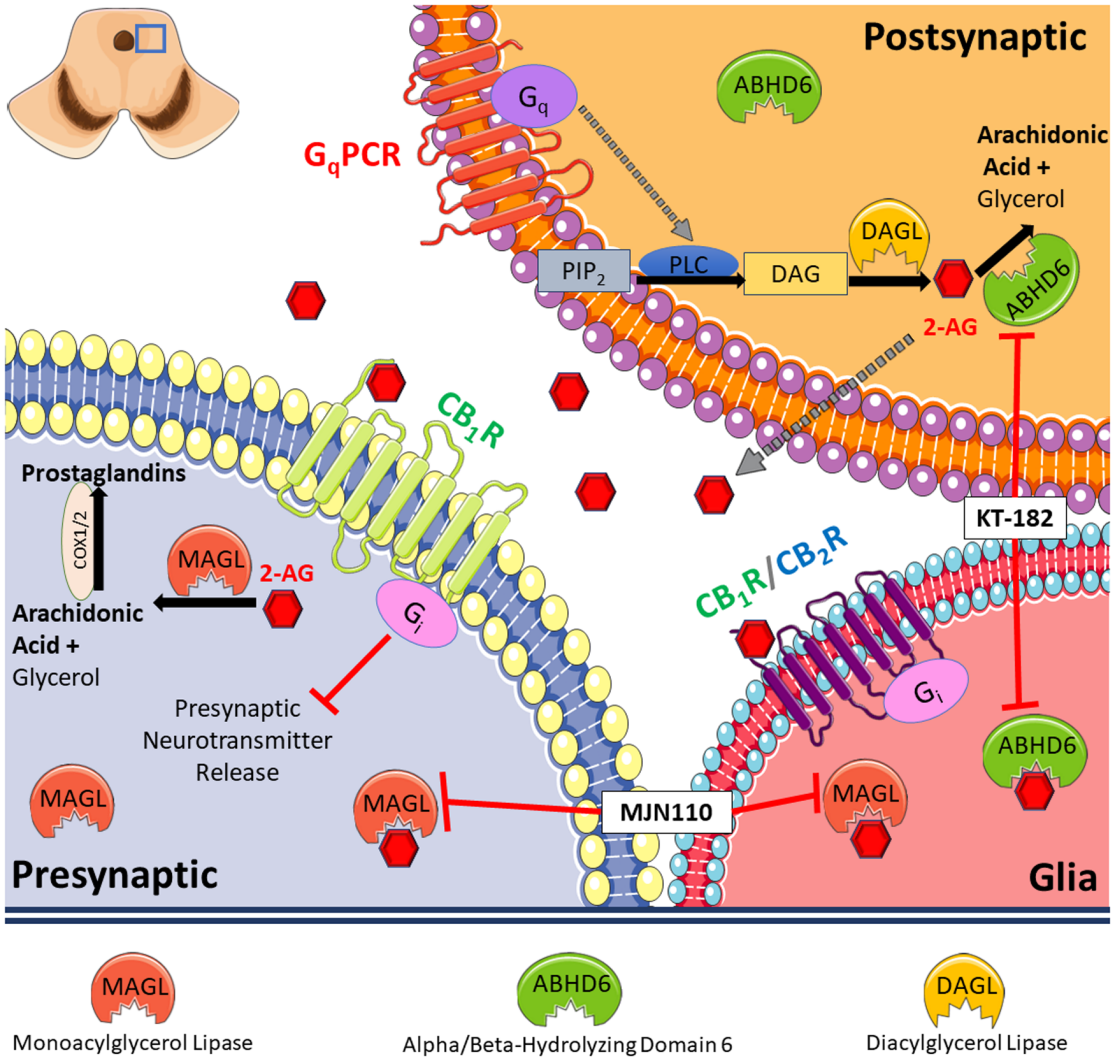
Summary of our findings. Our results indicate reduced level of 2-AG in PAG caused by cortical spreading depression (CSD) in female Sprague Dawley rats. This is accompanied by increased level of hydrolyzing enzymes, ABHD6 and MAGL, and induces a neuroinflammatory response. Pharmacological blockade of 2-AG degradation via inhibiting MAGL or ABHD6 both prevented and reversed periorbital allodynia associated with CSD induction.

Approximately 1/3 of migraineurs experience aura, a phenomenon linked to CSD and impaired PAG function (2, 4, 25, 26). We found that CSD events generated by cortical KCl injection in female rats reduce 2-AG levels in the PAG and elevate markers of neuroinflammation, supporting a role for ABHD6 and MAGL in CSD induction and maintenance. Pharmacological inhibition of MAGL or ABHD6 both prevented and reversed periorbital allodynia associated with CSD induction. Our study uncovers a mechanistic link between 2-AG hydrolysis and headache-like pain during CSD that can be therapeutically targeted (Figure 7).

It is well known that CSD induces neuroinflammation (23, 27). The expression of TNF*α*, IL-1β, and several members of interferon-mediated signaling is elevated after CSD induction (27). Mechanistically, 2-AG degradation leads to production of arachidonic acid, which is converted by cyclooxygenases to PGE_2_ (7). After CSD induction, we observed an increase in ABHD6 and MAGL detection, but not reductions in DAGL*α* detection, indicating increased 2-AG hydrolysis. This was coupled to reductions in 2-AG and increases in GFAP and PGE_2_. Notably, reduced levels of 2-AG occurred prior to PGE_2_ increases and at times of periorbital allodynia, in agreement with the PGE_2_ synthesis pathway. Together, these results support a role of rapid dysregulation of 2-AG in the PAG that facilitates PGE_2_ production, thus loss of 2-AG may mediate short term development of allodynia, while PGE_2_ mediated neuroinflammation facilitates the extended duration of periorbital allodynia observed following CSD induction (18). These results are consistent with other pain models showing that PGE_2_ in the PAG can facilitate hypersensitivity and that increased PAG functional activity corresponds to pain in migraine (25, 28). However, these results regarding ABHD6 and MAGL functional expression and the discrete circuits engaged will need to be confirmed in future studies, since total expression of these protein might not reflect their actual activity level.

Administration of ABHD6 and MAGL inhibitors significantly prevented and reduced CSD-induced periorbital allodynia, suggesting both a new strategy to treat headache pain and supporting prior reports with MAGL inhibitors in other headache models (12). Former work shows that administration of MAGL inhibitors, URB602 or JZL184 reversed established hyperalgesia in nitroglycerine-induced migraine models. Reduced neuronal activation in the ventrolateral PAG and Vc was also observed after MAGL blockade in the nitroglycerine migraine model (12). However, NO-donor induced periorbital allodynia was not reduced in MAGL knock-out mice (29). Therefore, further research is required to fully elucidate the role of MAGL in circuits implicated in headache-like pain. CB_2_R mediated the preventative and reversal effects of MAGL inhibition on CSD-induced periorbital allodynia. Notably, several recent Phase I clinical trials for the MAGL inhibitor ABX-1431 have shown promise in treating multiple neuropathic and pain-related disorders, including post herpetic neuralgia, diabetic peripheral neuropathy, and post-traumatic neuralgia though its clinical effects for migraine and headache have yet to be tested (30). The CB_1_R-independent nature of MAGL inhibition discovered in our model system prompts the development of such inhibitors to treat headache pain without producing psychotropic effects mediated by CB_1_R (31).

The effects of ABHD6 inhibition before CSD induction were cannabinoid receptor independent, whereas reversal of periorbital allodynia by ABHD6 blockade required CB_1_R. The cannabinoid receptor independence of ABHD6 inhibition in the prevention paradigm may be explained by how ABHD6 regulates activity-dependent increases of 2-AG levels, as ABHD6 degrades 2-AG prior to release (32). The difference of cannabinoid receptor dependency between ABHD6 and MAGL inhibition can also be explained by the different localization of the two enzymes, since MAGL is considered to express presynaptically, however ABHD6 localize in postsynaptic neurons (Figure 7). Furthermore, ABHD6 modulates the activity of multiple protein targets in addition to cannabinoid receptors, including GABA_A_ receptors, which may explain these findings (33). Alternatively, ABHD6 can metabolize mono-acylglycerols which may be elevated during ABHD6 inhibition that act at other receptor systems (e.g., GPR55, GPR119, PPAR) (34). Thus, the receptor(s) that mediate this preventative role of ABHD6 inhibition, as well as elucidation of cannabinoid-dependency/independency of MAGL and ABHD6 inhibitors remain to be identified.

At the cellular level, activation of CB_2_R by 2-AG controls microglial polarization and migration towards a resting state or anti-inflammatory response. Accordingly, reduction in 2-AG levels in PAG described is likely to reduce this drive and facilitate an M1, proinflammatory state (35, 36). Together, these studies suggest that reversal of CSD-associated allodynia by ABHD6 inhibition reflects control of neurotransmitter release, while MAGL blockade limits neuroinflammatory response. A recent review paper highlighted the role of neuroinflammation in the pathogenesis of migraine, beyond the classic neurogenic inflammation characterized by the release of neuropeptides such as CGRP and substance P (37). Peripheral levels of pro-inflammatory cytokines, such as interleukin-1β (IL-1β), IL-6, IL-8, and tumor necrosis factor-α (TNF*α*) were elevated in patients with migraine. Reduced levels of the anti-inflammatory cytokines, like IL-4 and IL-5 were also reported during migraine attack. Results obtained from animal models of headache also support that immunological responses associated with cytokines are involved in the mechanism of migraine, including enhanced production of several inflammatory cytokines, such as IL-1β, IL-6, and TNF-α during CSD. Endocannabinoids have been recognized as important players in mitigating neuroinflammation (38). Thus, controlling neuroinflammatory cascade by targeting endocannabinoid degradation can open a new avenue for future therapeutic options in migraine.

Limitations: Several limitations of this study exist. First, we administered enzyme inhibitors and receptor antagonists systemically. A recent study showed that the activity of MAGL in trigeminal ganglia is higher as compared to central sites, suggesting peripheral mechanisms of MAGL inhibition in alleviating migraine pain (39). Though peripheral activity of cannabinoid receptor antagonists is possible, SR141716 is shown to be brain penetrant, but similar studies have not been performed for SR144528 (40). SR144528 is, however, of consistent size with other molecules that demonstrate paracellular leak during CSD (18). Future studies are therefore warranted to examine microinjection of ABHD6/MAGL inhibitors and cannabinoid receptor antagonists into PAG to confirm central activity and specificity for cannabinoid receptor activity in the PAG. Secondly, there is a possibility that other brain areas that were not evaluated in the current study have impact on the endocannabinoid-dependent regulation of migraine pain, therefore investigation of additional brain regions is warranted in future studies. Third, the data suggesting that behavioral outcomes after MAGL inhibition occur via CB2 whereas ABHD6 inhibition leads to CB1R are not fully known; further studies investigating with respect to time and spatial organization are required. Lastly, all antibody-based assays are limited by the selectivity to the protein during normal and in post-translationally modified states; thus, the changes in antibody detection may reflect changes in antigen access, rather than changes in total expression.

## Data Availability

The raw data supporting the conclusions of this article will be made available by the authors, without undue reservation.
